# Acknowledgment to Reviewers of *Microorganisms* in 2021

**DOI:** 10.3390/microorganisms10020419

**Published:** 2022-02-11

**Authors:** Microorganisms Editorial Office

**Affiliations:** MDPI AG, St. Alban-Anlage 66, 4052 Basel, Switzerland

Rigorous peer-reviews are the basis of high-quality academic publishing. Thanks to the great efforts of our reviewers, *Microorganisms* was able to maintain its standards for the high quality of its published papers. Thanks to the contribution of our reviewers, in 2021, the median time to first decision was 14 days and the median time to publication was 33 days. The editors would like to extend their gratitude and recognition to the following reviewers for their precious time and dedication, regardless of whether the papers they reviewed were finally published:
Abate, GiuliaLaport, MarinellaAbbate, Jessica MariaLappa, IliadaAbd El-Aziz, Tarek MohamedLaranjo, MartaAbdel-Glil, MostafaLardé, HélèneAbdel-Rahman, Mohamed AliLarrea, EstherAbd-Elsalam, KamelLasik-Kurdyś, MałgorzataAbdeltawab, NourtanLaskowska, EwaAbia, Akebe Luther KingLastochkina, OksanaAbičić, IvanLatorre, EvaAbram, MajaLattorff, H. Michael G.Abugri, Daniel A.Laudadio, EmilianoAcedo, Jeella Z.Laura, Martinez-AlvarezAcosta-Jurado, SebastiánLauretani, FulvioAdam, WaśkoLavergne, Rose-AnneAdamakis, Ioannis-DimosthenisLavigne, Jean-PhilippeAdamčík, SlavomírLawal, OpeyemiAdamczyk-Popławska, MonikaLazar, Cassandre S.Adamska, EdytaLazar, CatalinAddepalli, BalasubrahmanyamLazarovits, GeorgeAdhikari, ArjunLe Coq, DominiqueAdhikari, BishnuLe Maréchal, CarolineAdhikari, Bishwo N.Lean, Fabian Z. X.Adhikari, RajanLee, Hee IlAdil, MohdLee, Je ChulAdkar-Purushothama, Charith RajLee, Kang-seokAdnan, MuhammadLee, Kyung-YilAdolfsson, AnnsofieLee, Marcia R.Afelik, SolomonLee, Meng-RuiAgnolucci, MonicaLee, NatuschkaAgudelo-Romero, PatriciaLee, Sang HeonAguiar, SandraLee, Sang-SukAhmad, AsrarLee, Yuan-TiAhmad, Siti AqlimaLegaz, IsabelAhmad-Nejad, ParvizLegione, AlistairAínsa, José A.Lehnherr, HansjörgAizpurua, OstaizkaLeifels, MatsAkbar, Sheikh Mohammad FazleLemos, Manuel L.Al, KaitLenhard, Justin R.Alao, John PatrickLeo, Jack ChristopherAlcázar-Fuoli, LauraLeone, Paolo MariaAldred, KatieLeoni, ClaudiaAlduina, RosaLepšová-Skácelová, OlgaAlessiani, AlessandraLetek, MichalAlex, AnoopLeu, Jiann-HorngAlexa, Elena-AlexandraLeuchtmann, AdrianAlexandropoulou, IoannaLeung, Ping-ChungAlexeevski, Andrei V.Levine, MindyAlexopoulos, AthanassiosLevitin, AnastasiaAleynova, Olga A.Levy, OrenAli, Hayssam M.Lewis, JenniferAli, Syed AzmalLi, BinAlice, RaffetinLi, CharlesAlisauskas, Ray T.Li, ChengleiAlizadeh, HosseinLi, DongmeiAlizargar, JavadLi, HaoAllen, AdrianLi, JieAllen, David M.Li, Ju-PiAllicock, Orchid M.Li, LeyuanAllison, KyleLi, LinAlmand, Erin A.Li, LuAlmazán, ConsueloLi, Mei-LingAlmeida, EduardoLi, MingjiAlvarenga S., Nelson L.Li, Po-HsienAlvarez, Anne MLi, QihanÁlvarez, María S.Li, ShucongÁlvarez-Mercado, Ana IsabelLi, YihangAlves, Carlos RobertoLi, Yong-caiAlvisi, GualtieroLianou, AlexandraAmalfitano, StefanoLiao, YifeiAman, M. JavadLiao, Yu-ChiehAmaro, FranciscoLiapikou, AdamanthiaAmbrosi, CeciliaLiaw, Shwu-JenAmbúr, Ole HermanLiburdi, KatiaAmillis, SotirisLicker, Monica SorinaAmils, RicardoLigat, GaetanAmmar, El DesoukyLikotrafiti, EleniAmmendola, SerenaLim, Hyun GyuAmoutzias, GrigorisLim, SangyongAmund, DanielLima, BeatrizAn, RanLin, Ching-HsuanAnacarso, ImmacolataLin, Chiu-YueAnagnostopoulos, Dimitrios A. Lin, Li-YinAnastasopoulou, AmaliaLin, Nai-ChunAnderson, AnneLin, QiangAnderson, Kirk E.Lin, Shih-ChaoAnderson, O. R.Lin, Yu-TeAndo, IstvanLinde, JörgAndonov, AntonLinden, SaraAndrade, José C.Lindkvist, KarinAndré, Marcos RogérioLindsay, DianeAndréani, JulienLindsey, John W.Andreani, Nadia AndreaLing, ChenAndreansky, SamitaLing, JiaxinAndreolli, MarcoLinial, MichalAndreoni, FrancescaLink, HannesAngelici, Maria CristinaLino-Neto, TeresaAnghel, LarisaLiolios, Konstantinos A.Angiolella, LetiziaLiordos, VasiliosAnja, KlančnikLiotti, FloraAnkrah, Alfred O.Liovic, IvicaAnstead, Gregory M.Liptáková, AdriánaAntonelli Incalzi, RaffaeleLiu, HaoyuAntonelli, AlbertoLiu, HuitaoAntonets, KirillLiu, JunAntonkiewicz, JacekLiu, Jun-HongAntonović, AlanLiu, LeiAntunes, JorgeLiu, Xiao-YongAnzola, Luz KellyLlama-Palacios, AranchaAonofriesei, FlorinLlenas-García, JaraApicella, AntonioLlorente, FranciscoApostol, Laura CarmenLo Giudice, AngelinaAragona, MariaLo, MichaelArana, InesLo, Shih-YenAranda, AgustínLobato-Marquez, DamianArango Duque, GuillermoLojkowska, EwaAraujo, RicardoLomakina, NataliaArce-Cervantes, OscarLondei, PaolaArcher, Nathan K.Long, MengxianArciszewski, MarcinLonghi, CatiaArdissino, GianluigiLoniewski, IgorArike, LiisaLopes, Ana Patrícia AntunesArita, MasanoriLopes, Ana RitaArita, MinetaroLopes, FilipaArnold, DawnLopez Galvez, FranciscoArora, GunjanLopez, Blanca R.Arrigoni, RobertoLopez-Jornet, PiaArteaga-Livias, KovyLópez-Medrano, RamiroArtyszak, ArkadiuszŁopieńska-Biernat, ElżbietaArukha, Ananta PrasadLorenzo-Morales, JacobArzamasov, AleksandrLos, MarcinAsakawa, MitsuhikoLoVerde, PhilAsh, AmandaLu, Ming-WeiAshe, MarkLu, YuyunAshour, HossamLuciani, MichelangeloAshrafi, SamadLuciani, MirellaAsman, MarekLuganini, AnnaAsnicar, FrancescoLuis, Ana SAspatwar, AshokLukes, JuliusAspri, MariaLunardi, MicheleAthanassiou, PanagiotisLund, MarianneAttila, GlatzLungaro, LisaAttili, Anna RitaLupi, Saturnino MarcoAubourg, SantiagoLuporini, PierangeloAuchtung, JenniferLüth, StefanAudigé, AnnetteLuttermann, ChristineAugustyniak, DariaLutzu, Giovanni AntonioAujayeb, AvinashLuzhetskyy, AndriyAulitto, MartinaLynch, CianAung, Meiji SoeMaack, ChristianAustin, BrianMacCarthy, EugeneAvendaño-Ortiz, JoséMacey, MichaelAvrani, SaritMacGregor, Barbara J.Awasthi, MayankaMaciver, SutherlandAwasthi, PraveenMacori, GuerrinoAwosile, BabafelaMacRae, Jean D.Ayala, JuanMacRaild, ChristopherAyayee, Paul AkwetteyMad’ar, MariánAzabou, SamiaMaddox, CarolAzam, Aa HaerumanMadeira De Carvalho, Luis ManuelAzinheira, Helena G.Madigan, Michael T.Azinheiro, SarahMaehara, ShojiAzzimonti, BarbaraMaekawa, HiromiBaba-Moussa, Lamine SaïdMaekawa, ShunBábíčková, JankaMagallanes, SergioBabiuk, LorneMagariños Ferro, BeatrizBabkin, Igor V.Magarlamov, Timur Yu.Baćmaga, MałgorzataMaggioli, Mayara F.Bae, Mi-JungMaguin, EmmanuelleBagi, AndreaMagurno, FrancoBagiu, Iulia-CristinaMahanty, SiddharthaBaglivo, IlariaMaicas, SergiBagyinszky, EvaMaisano, DomenicoBahadur, AliMaiti, SampaBahar, Ali AdemMajewska, AnnaBahl, Martin IainMajewska, MałgorzataBailly, BenjaminMajumder, KinjalBailoni, LuciaMak, Gannon C. K.Baindara, PiyushMalcangi, GiuseppinaBajaj, RuchikaMalfitano, Anna MariaBajanca-Lavado, Maria PaulaMałgorzata, ZiarnoBakaeva, MargaritaMalik, AdeelBalabanova, LarissaMaliszewska, Irena H.Balakirev, Evgeniy S.Malkin, SairahBaldin, ClaraMalli Mohan, Ganesh BabuBalestrini, Raffaella MariaMallo, NataliaBaliban, Scott M.Maloberti, AlessandroBanach, ArturMaloney, JennyBańbura, Marcin W.Malorny, BurkhardBanciu, HoriaMaltman, ChrisBanerji, AabirMan, AdrianBanks, LoriMandl, KarinBaptista, RodrigoManfrin, AmedeoBara, JeffreyMangiagalli, MarcoBarabasz, WieslawMangiaterra, GianmarcoBaraniak, AnnaMangone, IolandaBaratelli, MassimilianoMangot, Jean-FrançoisBarathikannan, KaliyanManilal, AseerBarbieri, GiuliaMann, Michael B.Barbieri, NicolleMann, Rachel A.Barbu Tudoran, LucianManni, MosèBarcena, CarmenManolescu, Loredana Sabina CorneliaBargiela, RafaelMansur, Ahmad RoisBar-Haim, ErezMantzoukas, SpiridonBaricz, AndreeaMarais, ArmelleBarinova, SophiaMaraolo, Alberto EnricoBarny, Marie-AnneMarascio, NadiaBarone, RobertoMarasco, GiovanniBarrasa, HelenaMarcacci, MauriliaBarreca, DavideMarchand, Patrice A.Barreca, SalvatoreMarchant, DavidBarrett, Janine L.Marchese, PietroBarros, JoanaMarciniak-Lukasiak, KatarzynaBarta, MarekMarcinnò, AndreaBartas, MartinMarczak, MałgorzataBartel, JürgenMareković, IvanaBarton, DiMaria Balestrini, RaffaellaBarton, Larry LMariani, Giulia MariaBartosik, Aneta AgnieszkaMariano, FilippoBartosik, DariuszMarimon, Jose MariaBartošová-Sojková, PavlaMarín, ClaraBarua, NilakshiMarincu, IosifBasaglia, MarinaMarino, RosannaBasco, LeonardoMariotti, CesareBashan, Luz E.Mark, NemethBastos, Reginaldo G.Markiewicz, Lidia HannaBates, PaulMarques, ClaudiaBatista, BrunaMarruchella, GiuseppeBatitucci, GabrielaMars, Ruben A.Batrinou, AnthimiaMarsili, EnricoBauza-Kaszewska, JustynaMartellos, StefanoBaymakova, MagdalenaMartin, FranckBazykin, Georgii A.Martinelli, MariannaBeale, DavidMartinez García, EstebanBeard, MatthewMartinez, Jose CBeauchamp, JonathanMartinez, OsvaldoBecchimanzi, AndreaMartínez-Hernández, SergioBeck, AndreasMartínez-Mota, RodolfoBedade, Dattatray K.Martínez-Rodrigo, AbelBeedessee, GirishMartinez-Sanz, ElenaBehar, AdiMartino, GiuseppeBehle, Robert WarrenMartino, Piera AnnaBehnam, Mira A.M.Martín-Rodríguez, Alberto J.Behnke-Borowczyk, JolantaMartins, IvoneBeier, Ross C.Martins, Larissa AlmeidaBejarano, AnaMartins-Loução, Maria AméliaBekal, SadjiaMartirosian, GayaneBekkat-Berkani, RafikMartorana, Alessandra M.Belanger, Faith C.Marutescu, Luminita GabrielaBeld, JorisMarverti, GaetanoBelk, MarkMas, AntonioBełka, MartaMascellino, Maria TeresaBelmonti, SimoneMashela, PhatuBelo, SilvanaMashima, IzumiBelov, Andrey A.Mashkova, Irina V.Beltran, María Del Carmen SánchezMasłoń, AdamBelyi, YuryMaslov, MikhailBelykh, OlgaMasłyk, MaciejBełżecki, GrzegorzMastanjević, KrešimirBender, KellyMastore, MaristellaBendjennat, MouradMastroleo, FeliceBenfield, David A.Mataragas, MariosBenítez-Mateos, Ana I.Matei, FlorentinaBenmerzouga, ImaanMatei, IoanaBenzertiha, AbdelbassetMateos-Hernández, LourdesBera, SoumenMathews, RanjivBerdalet, ElisaMathison, Blaine A.Berger, PetyaMatsumoto, ToshimiBerger, Ralf G.Matsuura, KatsumiBergh, ØivindMatsuzaki, RyoBerghoff, BorkMatteucci, ClaudiaBerleman, James E.Matyushenko, VictoriaBernacchi, SébastienMaurer, Anna C.Bernardes, NunoMauricio, IsabelBernardi, SaraMauriello, GianluigiBernaudin, Jean-FrançoisMaurin, MaxBernotienė, RasaMavrommatis, AlexandrosBerri, FatmaMaxwell, Patrick H.Bertaccini, AssuntaMayer, GhislaineBertea, CinziaMayfield, AndersonBertelloni, FabrizioMayo, BaltasarBerthold-Pluta, AnnaMayo-Prieto, SaraBertin, AurélieMazel, DidierBertrand, MartineMazurek-Popczyk, JustynaBertzbach, Luca DaniloMazzola, MarkBerzins, AivarsMazzone, PieraBęś, AgnieszkaMcCarthy, Lynda H.Betekhtin, AlexanderMcCarthy, PumtiwittBette, MichaelMcClelland, ErinBeuzón, Carmen R.McConville, MalcolmBevilacqua, AntonioMcGee, LesleyBeyi, Ashenafi F.McGregor, GlennBezirtzoglou, EugeniaMcgregor, NeilBhatt, PankajMcIntosh, E. David G.Bhattacharya, SomanonMcLean, RobertBhattacharyya, SanchariMcManus, Donald PBhowmik, DebsindhuMcMillan, ElizabethBi, JiaruiMcQueen, RachelBi, Qing-FangMcSkimming, Daniel IanBianco, FrancescoMcVicker, GarethBierle, CraigMcWhorter, AndreaBiernasiuk, AnnaMD Almeida, M CatarinaBiernat, Monika MariaMechrez, GuyBijle, Mohammed NadeemMedina, RoxanaBilli, DanielaMedo, JurajBinda, ElisaMehariya, SanjeetBindila, LauraMelekhina, E. N.Bingham, RichardMelis, AnastasiosBiolatti, MatteoMellies, JayBiondo, CarmeloMelville, StephenBiosca, Elena G.Mendes, Marta V.Birhanu, Biruk TesfayeMendes-Ferreira, AnaBischoff, MarkusMendes-Soares, HelenaBishop, KateMenéndez-Arias, LuisBitto, Natalie J.Menna-Barreto, Rubem Figueiredo SadokBiundo, AntonioMensi, MagdaBlagojevic Zagorac, GordanaMerabishvili, MaiaBlaha, ThomasMercado, Jesús M.Blanchard, AdamMerckx, AnaïsBlanc-Potard, AnneMertas, AnnaBlank, RalfMertz, GregoryBlasdel, BobMesquita, JoãoBlasdell, KimMessaritakis, IppokratisBlasi, ElisabettaMessenger, Andrew GuyBlauth de Lima, FernandaMessina, FrancescoBloom, Marshall E.Meto, AidaBlumer-Schuette, Sara E.Meurens, FrancoisBobek, JanMeyer, BenjaminBobeva, AneliyaMeyerhans, AndreasBochniarz, MariolaMézes, MiklósBoczek, Laura A.Meziti, AlexandraBogar, LauraMichail, MichailidisBohacz, JustynaMichalski, MirosławBohinc, KlemenMichel, Magdalena M.Bojanić, KrunoslavMichelet, LorraineBojinov, BojinMichels, PaulBoletis, IoannisMiddelveen, MarianneBolocan, AndreiMierzejewska, JolantaBolshoy, AlexanderMignolet, JohannBoncompagni, EricMihalca, Andrei DanielBonetta, SaraMihalj, MartinaBongiorno, DafneMikiyasu, SakanakaBordes, PatriciaMikuła, RobertBorges, ClarissaMiller, Aaron W.Borgmann, KathleenMiller, Catherine M.Boris-Lawrie, KathleenMiller, DarleneBorota, AnaMinami, MasaakiBorowicz, MarcinMiranda, LuisaBorsuk, GrzegorzMirete, SalvadorBortolami, AlessioMironeasa, SilviaBosilevac, Joseph M.Mishin, Alexander S.Bostik, PavelMishra, AbhishekBotelho, JoãoMishra, MeerambikaBoti, Michaela A.Mishra, SaurabhBotsaris, GeorgeMistou, Michel YvesBotterel, FrançoiseMitarai, SatoshiBouaïcha, NoureddineMitra, Amal K.Boucher, Yann FelixMitra, AvishekBoudreau, Paul D.Mitrea, LauraBouet, Jean-YvesMitsuyama, SusumuBourassa, Dianna VMittraparp-arthorn, PimonsriBourne, Christina R.Mizuta, KatsumiBoyer, PierreMnif, BasamaBrack, AndreMoawad, AmiraBrancaccio, MariaritaModesto, PaolaBranchu, PriscillaModi, AkshayBraun, BurgaMognetti, BarbaraBraun, RalfMohammad, ArifBréard, ÉmmanuelMohammadi-Aragh, MaryamBrejnrod, AskerMohan, GaneshBreves, GerhardMoiré, NathalieBreza, MartinMoiseenko, Konstantin V.Briani, FedericaMojica, KristinaBriant, LaurenceMoldovan, Oana TeodoraBRIDIER, ArnaudMoleleki, LucyBrígido, ClarisseMoles, Jean-pierreBrinkmann, HennerMolina, LázaroBrinks, ErikMollerup, Sørensen DannyBrito, Nuno V.Monaghan, Sean J.Britz, Margaret L.Moncalián, GabrielBrizić, IlijaMone, HélèneBrocato, RebeccaMonif, GillesBrocchi, MarceloMonokrousos, NikolaosBroecker, FelixMonteiro, SílviaBroenstad, AuroraMontemurro, MarcoBrogi, SimoneMontone, AngelaBrosnahan, Michael L.Montoya, AnaBrown, Ronald B.Montoya-Alonso, J. AlbertoBrownlee, Iain AMoon, Ki HwanBruchmann, SebastianMorais, Livia HeckeBrufau, JoaquimMoraru, Gail MiriamBrukner, IvanMoreau, EmmanuelleBrycki, BogumilMoreno Amador, María De LourdesBryła, MarcinMoreno, Andrea MickeBrzeszcz, JoannaMoreno, EstherBubak, IwonaMoreno, Miguel Á.Bubici, GiovanniMoreno, SandraBubnov, RostyslavMoreno-del Álamo, MaríaBuccheri, Maria-AntoniettaMoreno-Rojas, José ManuelBuchta, ChristophMorfin, NuriaBudaeva, Vera V.Mori, EmilianoBuddington, Randal K.Mori, HirotadaBueno De Mesquita, Clifton P.Mori, TetsushiBueno, EmilioMorikawa, KazuyaBugajski, PiotrMorón-López, JesusBugarin, AlejandroMorozov, Sergey Yu.Buil, Jochem B.Morozova, Vera V.Buitenhuis, Albert JohannesMorris, FayeBujdoso, GezaMorriswood, BrookeBukauskaitė, DovilėMortarino, MicheleBulaev, AleksandrMortzfeld, Benedikt M.Bulgariu, LauraMoșneguțu, Emilian FlorinBull, James J.Moss, Anthony G.Bullerjahn, GeorgeMossialos, DimitrisBülow, LeifMoszak, MałgorzataBültmann, HelgaMotherway, Mary O’connellBurchhardt, GerhardMotta, OrianaBursová, ŠárkaMottran, JeremyBuse, Helen Y.Motwani, MonaBushnev, Dmitriy A.Motyka-Pomagruk, AgataBusnelli, MarcoMouga, TeresaButaye, PatrickMoulick, AmitavaButenko, AnzhelikaMowbray, Catherine AButera, AndreaMowe, MaxineButiuc-Keul, Anca LiviaMozas-Moreno, JuanButkauskas, DaliusMrozik, AgnieszkaButler, JonathanMu, QinghuiButnariu, MonicaMucchetti, GermanoButtimer, ColinMucksová, JitkaBüttner, SabrinaMugetti, DavideByrd-Leotis, LaurenMukherjee, MaitreyeeByrne, AndrewMüller, BettinaByrne, MarcusMüller, JoachimCaballero, Raúl PérezMüller, Jochen A.Cabaret, JacquesMullié, CatherineCabezas-Cruz, AlejandroMullineaux, Conrad W.Caboň, MiroslavMunang’andu, Hetron MweembaCabral, HamiltonMunch, GarretCabrera, Jose Antonio GarciaMunder, AntjeCabrerizo, Marco J.Muniraju, MuraliCaceres, C. JoaquinMuñoz Atienza, EstefaniaČadež, NežaMunro, MoniqueCadilla, CarmenMuntyan, Maria SCaeiro, Maria FilomenaMurata, ShiroCafiero, Maria AssuntaMuravyov, MaximCafiso, AlessandraMurdaca, GiuseppeCaggiano, GiuseppinaMurdoch, David R.Cai, ShuoweiMuriel, JaimeCaito, SamuelMurkovic, MichaelCalabrese, Francesco MariaMurphy, NiamhCalcagnile, MatteoMurray, Shannon M.Calcutt, MichaelMurrell, ColinCalderón De La Barca, Ana MaríaMurugesan, KanagavelCalderón, Iván L.Mus, FlorenceCalero-Bernal, RafaelMusalgaonkar, SharmishthaCalisher, Charles H.Muszewska, AnnaCalla, JaesonMuszyńska, EwaCallewaert, ChrisMüthing, JohannesCama, JehangirMuxel, Sandra MarciaCampbell-Valois, François-XavierMyung, HeejoonCampeotto, IvanNa, Byoung-KukCamporesi, AnnaNaafs, BernardCampos, Maria Jorge GeraldesNadai, ChiaraCampos, Samuel K.Nadǎş, George CosminCanchaya, CarlosNagaoka, IsaoCandido, SaverioNagel, RaimundCanini, FabianaNagelkerke, NicoCano-Terriza, DavidNagy, BelaCantamessa, SimoneNaidu, PrenillaCanton, RafaelNaka, HiroakiCanuti, MartaNaka, ShuheiCao, HongnanNakamura, ShuichiCaparros-Martin, JoséNakanishi, AkiraCapasso, RaffaeleNakanishi, NorikoCapela, DelphineNalca, AysegulCappelletti, MartinaNale, JanetCappitelli, FrancescaNalepa, IrenaCapurso, LucioNandi, ShyamCaputo, LeonardoNanno, MasanobuCarabetta, Valerie J.Napiórkowska-Krzebietke, AgnieszkaCarbone, Dora AllegraNápoles, CaridadCardazzo, BarbaraNapoli, ChristianCardoni, MartinaNardini, RobertoCardoso, Danon ClemesNardo, Dario DiCardoso, FernandoNarita, Shin-ichiroCardoso, PauloNasar, FarooqCarestia, MariachiaraNasir, AbdulCarlier, JorgeNastuta, Andrei VasileCarlson, Hans KarlNatale, AldaCarnell, GeorgeNatale, GianfrancoCarotti, SimoneNath, ArijitCarr, Jillian M.Nathanailides, CosmasCarrano, Carl JNatu, RuchaCarrasco, JaimeNausch, MonikaCarroll, LauraNava, GerardoCarroll-Portillo, AmandaNavarro Garcia, FernandoCarter, NicolaNavarro, Juana MaríaCartmill, Andrew D.Navas, AlfonsoCaruso, GabriellaNavas, JesúsCaruso, PaolaNavascues, EvaCarvalho, EdmundNavolan, Dan-BogdanCarvalho, Lina A.S.Nawaz, FahimCasino, PatriciaNayak, Renuka R.Cassimos, DimitriosNazik, HasanCastagliuolo, IgnazioNeagu, SimonaCastelli, GermanoNeelakantan, PrasannaCastiglione, RobertoNeely, Melody N.Castiglioni, BiancaNeffe-Skocińska, KatarzynaCastillo Lopez, EzequiasNegrea, PetruCastillo, PabloNegut, IrinaCastro, Isabel V.Nemes, KatalinCastro, José Javier FernándezNerlich, AndreasCastro, Juan C.Neumann, BerndCatão, Elisa C. P.Newton, NataleeCavallero, SerenaN’Gouemo, ProsperCaviglia, Gian PaoloNguyen, Anh DucCebrián Castillo, RubénNguyen, Minh-ThuCeccanti, CostanzaNguyen, Quang D.Ceci, AndreaNguyen, Van KhanhCelandroni, FrancescoNicholas, RobinCelec, PeterNichols, Robert G.Cellina, M.Nicolai, EleonoraCellini, AntonioNicoletti, LoredanaCenci, ElioNicosia, AldoCenian, AdamNielsen, SigneCerbo, Alessandro DiNiemczyk, StanisławČernáková, LuciaNighot, MeghaliCerqueira, LauraNiklasson, BoCerrato, AndreaNikolich, MikeljonČerveň, JiříNikulin, AlexeyCesbron, SophieNiqueux, ÉricChagas, Carolina Romeiro FernandesNishida, HiromiChaintoutis, Serafeim C.Nishimura, AkiraChakankar, Mital V.Nishiyama, MitsueChakraborty, AmritaNissapatorn, VeeranootChalmers, GabhanNizami, Abdul-SattarChambel, LéliaNizza, AntoninoChamlagain, BhawaniNóbrega, CláudiaChandrasekaran, MurugesanNocera, Francesca PaolaChang, Kwang PooNoda, MasafumiChang, PengxiangNoel, Samantha J.Chang, Tsung-HsienNoll, MatthiasChanway, ChristopherNouioui, ImenCharoensutthivarakul, SitthivutNovak, JasnaChateau, AliceNovello, GiorgiaChaturvedi, DeeptiNovotna, AlzbetaChaudhary, Dhiraj KumarNowak, HartmuthChauhan, NikhilNowakiewicz, AnetaChaves, Luis FernandoNowakowicz-Dębek, BożenaChavez-Santoscoy, Rocio AlejandraNowakowska, Justyna AnnaChedea, Veronica SandaNsanzabana, ChristianChekanov, KonstantinNtaikou, IoannaChen, Chien-ChinNtemiri, AlexandraChen, Chih-ChiehNucera, EleonoraChen, ChuanNunes, MónicaChen, FengNurjadi, DennisChen, FengjuiNutricati, ElianaChen, Jen-TsungNyman, DagChen, Jiann-ChuObalová, LucieChen, LianminOborník, MiroslavChen, Ming-JuObrenovich, Mark E. M.Chen, Ying-ChiehO’Callaghan, DavidCheng, Hong ShengOczkowicz, MariaCheng, LiangOda, MasatakaCheng, Shih-LungOdemuyiwa, Solomon O.Cheng, ZishuoOduor, Joseph Michael Ochieng’Chenoll, EmparOey, MelanieCheong, Hae SukOgashawara, IgorCheptsov, VladimirOgawa, AkikoChernyh, Nikolay A.Ogawa, MasahiroChetverikov, SergeyOgihara, JunChevillard, ChristopheOgórek, RafałChianese, AnnalisaOgrodowczyk, AnnaChiang, Chih-PoOh, ChulhongChiang, Hsin-IO’Halloran, ConorChiang-Ni, ChuanOhtomo, RyoChiarelli, LaurentOishi, KenjiChiarini, LuigiOishi, TomohiroChieffi, DanieleOja, JaneChiellini, CarolinaOkamoto, ShigefumiChifiriuc, CarmenOkińczyc, PiotrChifiriuc, Mariana CarmenOkoń, AnnaChigbu, De Gaulle IOkura, MasatoshiChinivasagam, Helene N.Olaya Abril, AlfonsoChiou, Chien-ShunOldfield, NeilChistoserdova, LudmilaOliveira Souza, Ana CamilaChmielewska, JoannaOliveira, ManuelaChmielik, Lechosław PawelOliveira, Pedro H.Cho, Do-YeonOlivotto, IkeChoi, Jong-YunOlli, KalleChoi, JungilOlmos, AntonioChoi, Ki ChoonOlofsson, MalinChoi, Nag-ChoulOndrušková, EmiliaChoi, Woo JuneO’neill, Catherine A.Chong, RogerOngerth, JerryChou, Lan-SzuOnyedibe, Kenneth I.Chow, Pizza Ka YeeOnyilagha, ChukwunonsoChow, Yen-HungOrefice, IdaChowdhury, Chanchal SurOrfanoudakis, MichailChristensen, HenrikOrive, Alejandro GonzálezChristie, GrahamOrlando, GabriellaChristina Pillon, MonicaOrlić, SandiChristodoulides, MyronOrman, MehmetChrzanowski, ŁukaszOrruño, MaiteChua, Eng GuanOrsini, MassimilianoChubarov, AlexeyOrtega, Joseph ThomasChung, KwiMiOrusa, RiccardoChung-Hsiung, HuangOsek, JacekChurchward, ColinOshkin, Igor Y.Ciancio, AurelioOsório, Nuno S.Cid, DoloresOstash, BohdanCieraad, EllenOsterman, AndreasCiesielski, SlawomirOtaka, JunnosukeCieslak, AdamOtalora, MonicaCilia, GiovanniOtani, SariaCinek, OndrejOtlewska, AnnaCiobanu, MadalinaOts, KatriCipak, LubosOtulak, KatarzynaCiura, KrzesimirOulghazi, SaidCizek, AloisPacciarini, Maria LodovicaClark-Curtiss, Josephine E.Pacholewska, AlicjaClegg, Simon L.Pacífico, CátiaCocco, RaffaellaPacurari, MaricicaCoelho, AdrianoPaczkowski, JonCohen, RegevPadkina, Marina V.Cojoc, Lucia-RoxanaPadra, MédeaColasante, ClaudiaPadrão, JorgeCole, JeffPadzik, MarcinColin, RemyPagán, IsraelCollart, Martine A.Page, MalcolmColussi, SilviaPajdak-Czaus, JoannaCompton, AlexPajkrt, DasjaComte, KatiaPal, DhimanConnor, Edward F.Palenzuela, OswaldoConsole, LaraPalkovics, LászlóConstantinos, EhaliotisPalla, FrancoContaldo, MariaPalma, MargaridaContaldo, NicolettaPalma, Paulo J.Contardi, MarcoPalmer, AndrewCook, CharlesPalmieri, FerdinandoCook, RogerPalop, AlfredoCooper, Christopher D.O.Pampuro, NiccolòCooper, KerryPan, MeichenCooper, Lee W.Pan, XiaolingCopolovici, Dana MariaPanda, SantoshCordero-Bueso, GustavoPandit, SantoshCornelis, PierrePandoua Nekoua, MagloireCorona, MiguelPandur, EdinaCorreia, MariaPang, BoCorrente, MarialauraPanikov, NicolaiCorretto, ErikaPantea, StelianCorsaro, DanielePaolucci, MarinaCortegiani, AndreaPapa, GuidoCortes-Perez, Naima G.Papademas, PhotisCosoveanu, AndreeaPapadimitriou, KonstantinosCosseddu, Gian MarioPapadopoulos, GeorgiosCosta, DamienPapagiannitsis, Costas C.Costa, MônicaPapaianni, MarinaCotârleț, MihaelaPapanikolaou, EleniCouvin, DavidPapanikolaou, SeraphimCox, LauraPapatheodorou, EfiCraciun, NicolaiPaploski, IgorCrawford, Emily D.Pappa, Olga D.Crecchio, CarminePappritz, KathleenCrespo-Rivas, Juan CarlosPapp-Wallace, Krisztina M.Crevecoeur, SophiePaquin-Proulx, DominicCrisan, LuminitaParada, ClaudiaCristiano, GiuseppeParamythiotis, SpirosCristino, SandraParaschiv, MariusCroft, LarryParente, EugenioCrognale, SimonaPark, DanielCrook, BrianPark, Eun JeongCRUZ, IsraPark, HyunCruz, JoanaPark, Hyun-sunCubero, JaimePark, Hyun-WooCuellar-Gempeler, CatalinaPark, Jong-TaeCui, HaiyangPark, Myeong SooCulebras, EstherPark, Se ChangCull, BenjaminPark, Si JaeCunha, EvaPark, Soo JeCuong, NguyenPark, YoungjinCutone, AntimoParkar, Shanthi G.Cuypers, BartParra-Flores, JulioĆwieląg-Piasecka, IrminaParthasarathy, AnutthamanCyplik, PawełPasechnik, TatyanaCzajkowski, RobertPasin, FabioCzernek, JiříPasini, Erica M.Czernel, GrzegorzPassera, AlessandroCzupryna, PiotrPassero, FelipeDa Rosa, Joao AristeuPaster, Bruce JDa Silva, Luís C. N.Pastor, Sandra GarcésDabert, MirosławaPastore, Glaucia MariaDąbrowska, JoannaPatching, Simon GD’Acquisto, FulvioPatel, Jai SinghDaghio, MatteoPatila, MichaelaD’Agostino, PaulPatini, RomeoDahanayake, P.S.Patiño-Navarrete, RafaelDahiya, RajivPatkowska, ElżbietaDahiya, SatinderPatra, Jayanta KumarDal Bello, MartinaPatsios, SotirisDall, DavidPatterson, IanDaly, Seth M.Paudel, AtmikaDamaziak, KrzysztofPaukov, AlexanderDame-Korevaar, AnitaPaul, AlokD’Amico, FedericaPaulette, LauraDanforth, David N.Paulino-Lima, Ivan GláucioD’Angeli, FlorianaPaulitsch-Fuchs, AstridDangi, TanushreePaulo, CarlosDanilenko, Daria M.Paulsen, PeterDanne, CamillePauvolid-Corrêa, AlexDanshiitsoodol, NarandalaiPavelko, Kevin D.Darvishi, FarshadPavli, FoteiniDatta, RahulPavlík, IvoD’auria, EnzaPavlović, HrvojeDavenport, Colin F.Pawlik, AnnaDavid Vejarano Mantilla, RicardoPawlos, MałgorzataDavid, JohnPawłowska, AgnieszkaDavidovich, NadavPayami, HaydehDavidson, IritPayot, SophieDavies, Bryan WilliamPayot-Lacroix, SophieDavolos, DomenicoPeces, MiriamDavydenko, KaterynaPech-Cervantes, Andres AlfredoDawen Yu, EstherPeck, RonaldDawood, Mahmoud A.O.Pecyna, PaulinaDay, Andrew S.Pedelacq, Jean-DenisDayal, NeetuPedonese, FrancescaDe Aguiar Cordeiro, RossanaPedraza Chaverri, JoséDe Alteriis, ElisabettaPedro, SóniaDe Bonis, SalvatorePeguda, Hari De Castro, SoniaPeiris, MadushaDe Groot, ArjanPeixe, LuísaDe Jesus, MagdiaPellegrini, MarikaDe La Cruz, Armah A.Pellegrino, EmilioDe La Rua Domenech, RicardoPena, Maria MarjoretteDe La Torre, Beatriz G.Peng, Chi-ChungDe la Torre, Juan C.Peng, Kou-ChengDe La Torre, Maria ÁngelesPeng, MaoDe Lana, MartaPensec, FloraDe Luca, DanielePenttinen, ReettaDe Lurdes Dapkevicius, MariaPénzes, Judit J.De Masi, LuigiPepi, MilvaDe Paola, DomenicoPerchec Merien, Anne-MarieDe Pasquale, IlariaPereira, CarlaDe Sanctis, Juan BautistaPereira, Carlos DiasDe Winde, J.H.Pereira, ClaraDea-Ayuela, MaríaPerelomov, Leonid V.Deamici, KricellePérez, JuanaDeback, ClairePérez-Arques, CarlosDebRoy, ChitritaPérez-Cataluña, AlbaDec, MartaPérez-Cobas, Ana ElenaDeepe, GeorgePérez-Díaz, Ilenys M.Dekker, MarloesPérez-García, FelipeDel Gallo Di Roccagiovine, Maria MaddalenaPérez-Sánchez, RicardoDel Gallo, MddalenaPernek, MilanDelbac, LionelPerner, JanDelfraro, AdrianaPerry, John D.Delgado, RafaelPertile, GiorgiaDelgado-Huertas, AntonioPessela João, BenevidesDell’Anno, MatteoPestov, DimitriDellière, SarahPetera, KarelDelorme, ChristinePeterson, Karianne LievaartDemanèche, SandrinePetinaki, EfthimiaDemey, BaptistePetříčková, KateřinaDenesvre, CarolinePetrikovszki, RenátaDeng, FeilongPetryszak, PrzemyslawDeng, LiPezzella, CinziaDenkova-Kostova, Rositsa StefanovaPfiffner, SusanĐermić, EdytaPhanse, YashdeepDeshpande, GauraviPhilipp, BodoDeviatkin, Andrei A.Piatek, JacekDevkota, Hari PrasadPiatti, GabriellaDey, Debasish KumarPick, FrancesDey, PriyankarPickering, RaeleneDhakal, SantoshPicó-Monllor, José AntonioDhar, NeerajPiekarska, KatarzynaDharmaraj, RajmohanPierangeli, AlessandraDhowlaghar, NitinPierce, ElizabethDi Cosola, MichelePietrasanta, CarloDi Gennaro, FrancescoPigeault, RomainDi Giallonardo, FrancescaPignataro, GiuliaDi Marco, RobertoPilarska, Agnieszka A.Di Profio, FedericaPiłsyk, SebastianDi Stasio, DarioPinacchio, ClaudiaDi Stefano, MariantoniettaPincus, SethDiakou, AnastasiaPinel-Marie, Marie-LaureDiamond, GillPinsino, AnnalisaDiaz Fernadez, PabloPinto, BarbaraDiaz-Amaya, SusanaPinto, LorisDieci, GiorgioPinzari, FlaviaDietrich, IsabellePiot, NielsDilfuza, EgamberdievaPircalabioru (Gradisteanu), GratielaDimitrellou, DimitraPires, JoãoDimmock, NigelPisano, IsabellaDimopoulou, DimitraPisklak, Dariusz MaciejDimova, IvankaPistocchi, Anna SilviaDing, ChangPiu, SahaDing, ShunpingPiwowarek, KamilDinkla, InezPizzorno, Mario AndresDinte, ElenaPlacido, AntonioDiogo, PatriciaPlatonov, Alexander E.Dionisio, GiuseppePławińska-Czarnak, JoannaDirkx, LauraPleskova, SvetlanaDiSalvo, SusannePlotnikov, AndreyDishon, ArnonPobiega, KatarzynaDišlers, AndrisPodleśny, JanuszDittami, Simon M.Pogoreutz, ClaudiaDiţu, Lia MaraPolak-Berecka, MagdalenaDixit, PoojaPolčic, PeterDkhar, Hedwin KitdorlangPoldmaa, KadriDługosz, EwaPolimeno, LorenzoDobosz, RenataPoluéktova, Elena U.Dobrescu, AmadeusPolz-Dacewicz, MałgorzataDobrovolny, Hana M.Pomastowski, PawełDobrowolski, AdamPomel, SebastienDobrowolski, PiotrPonnusamy, LoganathanDolan, Brian P.Pontrelli, SammyDolce, DanielaPopa, Gabriela LoredanaDolegowska, BarbaraPopa, Mircea IoanDoligalska, MariaPopescu, Cristina Raluca Gh.Domann, EugenPopluechai, SiamDomenech, AnaPopova, Alexandra A.Dominguez, Delfina C.Porollo, AlekseyDomokos, ErzsébetPortugal, AntonioDonachie, StuartPotaczek, Daniel P.Donadeu, MeritxellPotekhin, AlexeyDonaire, LiviaPotgieter, NatashaDonaldson, JanetPoulicek, Mathieu E. A.Dong, KePous Rodríguez, NarcísD’Onofrio, GiuseppePoveda, JorgeDos Santos Dias, LucasPowell, MarkDouda, OndřejPoyntner, CarolineDoulberis, MichaelPozzi, CeciliaDrahovská, HanaPrabhala, Bala KrishnaDrlica, KarlPrabhu, Ashish ADrosinos, EleutheriosPradeep Ram, Angia SriramDruszczyńska, MagdalenaPrado, SamiraDrzewiecka, KingaPraeg, NadineDuan, KangminPresentato, AlessandroDuarte Ritter, CamilaPrete, RobertaDuarte, AidaPrévost, JérémieDuarte, MargaridaPrevots, RebeccaDubé, Caroline EPrice, PatriciaDubé, MathieuPrice-Carter, MarianDubey, Ashutosh P.Prieto, MiguelDubey, Jitender PPrigigallo, Maria IsabellaDuda-Chodak, AleksandraPristaš, PeterDuda-Madej, AnnaPriyadarshini, MedhaDudás, ZoltánProbert, ChrisDudkin, Semyon V.Probst, MaraikeDufossé, LaurentProcopio, Francesco AndreaDugar, SiddharthProctor, RichardDuggan, JackiePronin, Alexander V.Duliu, Octavian G.Proszkowiec-Weglarz, MonikaDumic, IgorPrzekop, RobertDumitru, Croitoru MirceaPsaroulaki, AnnaDumke, RogerPucciarelli, SandraDumoux, MaudPuchkov, EvgenyDunaj, JustynaPuente-Rivera, JonathanDunisławska, AleksandraPuray-Chavez, Maritza N.Dunn, JohnPurdy, Michael A.Duprat, ElodiePurkrtová, SabinaDutta, SanjuctaPutonti, CatherineDüzgüneş, NejatPuzanskiy, RomanDyall-Smith, MikePyrri, IoannaDzidic, AlenPyzik, AdamDziejman, MichellePyz-Łukasik, RenataDziendzikowska, KatarzynaQian, WeiEbelt, Nancy DanielleQin, HuaEdit, FarkasQu, ZhishuaiEdwards, Adrianne N.Quaglio, FrancescoEfremenkova, Olga VQuan, Fu-ShuiEgamberdieva, DilfuzaQuang, NgyenEgerton-Warburton, LouiseQuante, MichaelEgyed, LászlóQuéré, GaëlleEid, AhmedQuinto, Emiliano JoséEl Alaoui, HichamRab, AndrásElaswad, AhmedRABAH, HouemEldridge, SandyRabemanolontsoa, HarifaraEl-Duah, PhilipRadadiya, AshishEliopoulos, Panagiotis A.Radcliff, Fiona JaneElitas, MeltemRadoslavov, GeorgiElkins, KellyRadu, Nicoleta NicoleElla, BhagyarajRadzki, RadosławEllis, ChristopherRafieenia, RaziehElnekave, EhudRagusa, AndreaElolimy, Ahmed A.Rahlff, JaninaElsayed, Ahmed MostafaRahman, M. AzizurElshamy, AbdelsamedRahnavard, GholamaliEmilio, MariagraziaRai, NavneetEmmerstorfer-Augustin, AnitaRaina, SatishEngelmann, JuliaRaja, Huzefa A.Ercibengoa Arana, MariaRajagopalan, KrithikaErgal, IpekRajeeve, KarthikaEriani, GilbertRajendran, Ranjith KumarErmolenko, Ermolenko E.I.Rajkumar Singh, KalraErsfeld, KlausRajput, Vishnu D.Ertl, ReinhardRakov, A. V.Escudero-Pérez, BeatrizRamalingam, SundarEsposito, FrancescoRamanathan, PalaniappanEspuelas, SocorroRamirez, Jose LuisEsteban-Zubero, EduardoRamírez-Paredes, Jose GustavoEstensoro, ItziarRampacci, ElisaEstrada-Peña, AgustínRampazzo, Rita De Cássia PontelloEtebari, KayvanRamsay, JulianaEto, Silas FernandesRanalli, GiancarloEzra, DavidRanallo, RyanFabiszewska, AgataRando, Maria MargheritaFacey, PaulRangel, Gabriel WFagerquist, CliftonRangel-Huerta, Oscar DanielFakhri, OmidRanjan, KishuFalagan, CarmenRaoult, DidierFalconi, MaurizioRaposo, RosaFalkinham, Joseph O. IIIRasheduzzaman, MdFarcasanu, Ileana C.Rasiukevičiūtė, NeringaFaria, Jorge M. S.Rasmussen, Hanne N.Farina, AntonellaRatajczak, MagdalenaFarkas, AncaRather, GulamFarzan, VahabRather, Irfan A.Farzaneh, VahidRathod, SanjayFattorini, LanfrancoRathore, AtulFederici, MassimoRatiu, AndreeaFederici, SaraRaval, YashFehér, EnikőRaven, John AlbertFelgueiras, HelenaRavin, Nikolai V.Feliciano, JoanaRavindran, RajeevFendrihan, SergiuRay, RanjitFeng, HuapengRay, SamriddhaFerella, FrancescoRazafindralambo, Hary L.Fernandes, Paulo A.Razim, AgnieszkaFernandez, JessieRazvan-Cosmin, PetcaFernández, LucíaRebelos, EleniFernández-Coll, LlorençRedder, PeterFernández-Tomé, SamuelRedrejo Rodriguez, ModestoFerrante, PasqualeReese, Aspen T.Ferraz, Maria PiaRégnier, MarionFerreira, Ana CristinaReich, AdamFerreira, Carlos Miguel HenriquesReid, DeborahFerreira, MartinsReid, St PatrickFerreira, NelsonReisser, CelineFerreira-Paim, KennioRemenant, BenoitFerrigo, DavideRemm, MaidoFeuilloley, Marc G.J.Rendueles, ManuelFidalgo, Liliana G.Renu, SankarFigarella, KatherineRequena, Jose MaríaFigueiredo, AndreiaResch, BernhardFilion, MartinRešetar Maslov, DinaFilip, RafałRey, ThomasFilipič, BratkoReyes-Ramírez, FranciscaFinore, IlariaRibeiro, ApoenaFiorenza, Salvatore SamRibeiro, Rita Cláudia CardosoFirrincieli, AndreaRibeiro-Barros, Ana I.Fischer, Egil Andreas JoorRicchi, MatteoFiskal, AnnikaRicci, AnnalisaFisunov, GlebRicciardelli, AnnaritaFit, NicodimRichards, NgaioFlorek, EwaRichert, AgnieszkaFlorek, MariuszRichi, PatriciaFlores, HeatherRichner, JustinFlores-Chávez, María DelmansRichter, LukaszFodor, PéterRichtera, LukášFol, MarekRidolfo, Anna LisaFolco, Hernan DiegoRiedle-Bauer, MonikaFöldvári, GáborRijpkema, SjoerdFong, KarenRinaldi, ElenaFonseca, KevinRinder, MonikaFormenti, NicolettaRindi, LauraFort, IsabelRingqvist, EmmaFouladkhah, AliyarRios-Covian, DavidFraaije, BartRiska, PaulFrancavilla, RuggieroRiva, GiuseppeFranceschi, PieroRivas Franco, FedericoFranciosa, GiovannaRivas-Marin, ElenaFrancisco, Alexandra E.Rivers, AdamFranco, Carlos M.Rivoal, KatellFranco, DomenicoRizzo, CarmenFrassanito, AntonellaRobak, MałgorzataFréalle, EmilieRobert-Gangneux, FlorenceFreestone, PrimroseRoberts, MarilynFreimanis, GrahamRobertson, Gregory J.Freimoser, FlorianRobeson, MichaelFrías-De-León, María GuadalupeRobinson, Derrick R.Friedman, Elliot S.Roby, Justin A.Frimodt-Møller, NielsRocchetti, GabrieleFrye, JonathanRocchigiani, GuidoFuchs, BenjaminRocha Faustino, Ana IsabelFuchs, MarcRodamilans, BernardoFuentes, Màrius V.Rodrigo, ChaturakaFujii, ShoRodrigue, AgnèsFujita, MasakiRodrigues, ArmandaFujiwara, Ricardo ToshioRodrigues, CarlaFukiya, SatoruRodrigues, Clara F.Fukuda, AkiraRodrigues, FranciscaFukui, HirokazuRodrigues, JoanaFukushima, KentaroRodrigues, Mário S.Fukushima, KiyoharuRodriguez, ChristopheFurmańczyk, Ewa M.Rodriguez-Llorente, Ignacio D.Furmanek-Blaszk, BeataRodríguez-López, PedroFurneri, Pio MariaRodriguez-Morales, OliviaFurtak, KarolinaRodríguez-Villodres, ÁngelFurukawa, KenjiRodziewicz, JoannaGabriel, IwonaRohde, John R.Gabriele, MorenaRojas-Lopez, MaricarmenGaeta, Giovanni BattistaRojo, Maria AngelesGaffney, Erin M.Rokosz, KrzysztofGajdoš, PeterRolla, GiovanniGajdosik, Martina SrajerRomán López, PabloGala, Rikhav P.Romano, Giovanni LucaGalachyants, Agnia D.Romano, JuliaGalal, LokmanRomano, M. CarmenGalan Ladero, Maria AngelesRomano, PatriziaGalan, Ana-MariaRomantschuk, MartinGalanis, AlexisRomero Noguera, JulioGallart, MartaRomero, Antonia MaríaGallert, ClaudiaRoncada, PaolaGallier, SophieRoncero-Ramos, BeatrizGallo, GaetanoRong, Chan KuanGallo, GiovanniRoques, ChristineGallo, MonicaRosales-Mayor, EdmundoGallo, SimoneRosana, Albert Remus R.Gamalero, ElisaRosberg, Anna KarinGambino, Caterina MariaRoscetto, EmanuelaGan, Hin HarkRoscini, LucaGancheva, Galya IvanovaRoss, ElizabethGanesan, A.Rossi, FrancaGanter, MarkusRougeaux, ClemenceGao, RuiminRousseau, GuyGaraizar Candina, JavierRousseaux, SandrineGarbayo, InésRout, Simon PGarcia, MagaliRoy, AmitGarcía-Álvarez, LaraRoy, Jean-PhilippeGarcia-Descalzo, LauraRóżalska, BarbaraGarcía-Mina Freire, José MaríaRóżycki, MirosławGarcía-Ríos, EstéfaniRuan, ZhiGarcia-Rubio, RocioRuban, DmitryGarcía-Suárez, María Del MarRudzki, LeszekGargari, GiorgioRuffin, FeliciaGarman, Lori D.Rugna, GianlucaGarrido, CarlosRuiz Jimenez, AntonioGarrido-Fernández, AntonioRuntuwene, Lucky RonaldGarrido-Maestu, AlejandroRusso, BrianGasmi, LeilaRusso, David AGasparatos, DionisiosRužauskas, ModestasGaspersic, RokRyabchikova, Elena I.Gastineau, RomainRybczyńska-Tkaczyk, KamilaGaul, DavidRyberg, MartinGayà-Vidal, MagdalenaRychter, Anna MariaGazi, Anastasia D.Rysiak, AnnaGazzonis, Alessia LiberaRząsa, AnnaGeddes-McAlister, JenniferRzewuska, MagdalenaGekenidis, Maria-TheresiaSaade, AnastasiaGell, David A.Sabater-Muñoz, BeatrizGenestet, CharlotteSablé, SophieGenovese, CarloSaccà, Maria LudovicaGentili, RodolfoSacks, David LawrenceGerdol, MarcoSadarangani, ManishGeva-Zatorsky, NaamaSadeghianmaryan, AliGhafar, AbdulSádlová, JovanaGhenea, Alice ElenaSaffioti, CarolinaGherman, Calin MirceaSagova-Mareckova, MarketaGhita, SimonaSaha, RanajayGhosh, ArnabSahgal, PranshuGhosh, PrithwiSahl, Jason W.Ghosh, SourishSahni, Sanjeev K.Ghoul, MelanieSaid-Al-Naief, NasserGiacobbe, Daniele RobertoSaint-Fort, RogerGiacomelli, AndreaSaito, MasanoriGiammanco, AnnaSajewicz, JoannaGianfredi, VincenzaSakamoto, HirokazuGianotti, AndreaSakamoto, MitsuoGiantsis, Ioannis A.Saksena, NitinGibellini, DavideSakurai, KenichiGibson, GlennSalaheen, SerajusGil, RosarioSalamon, DominikaGilbert-Girard, ShellaSalant, HaroldGilmour, MatthewSalari, AlinaghiGilot, PhilippeSalbitani, GiovannaGiordani, BarbaraSaleem, MuhammadGiorgio, SelmaSalerno, CarloGiraldo, Martha CeciliaSalmanton-García, JonGiraud, GuillaumeSalmerón, IvanGirones, NuriaSalvador, AndreiaGiudice, AmerigoSalvato, MariaGiuffrè, MauroSamardžija, MarkoGiurg, MirosławSamelis, JohnGkizi, DanaiSamhan-Arias, AlejandroGlaizot, OlivierSampaio, AnaGlass, N. LouiseSampaio-Marques, BelémGlendinning, LauraSampedro, InmaculadaGloer, JamesSamylina, Olga S.Gniewosz, MałgorzataSanabria, JanethGobin, IvanaSánchez, IsraelGodfrey, Henry P.Sanchez, SophieGodoy-Vitorino, FilipaSánchez-Gorostiaga, AliciaGoeres, DarlaSándor, Attila D.Goeser, FelixSandra F., BorgesGoga, MichalSanjuán, JuanGoic-Barisic, IvanaSantacroce, LuigiGoldford, Joshua E.Santaeufemia, SergioGolomazou, EleniSantamaría, Ramón I.Gomes, Ana CordeiroSantana, Andrés GonzálezGomes, Ana RitaSantella, BiagioGomes, FabioSantoro, FrancescoGómez Bolea, AntonioSantos, FernandoGómez Pinchetti, Juan LuisSantos, RicardoGómez Villalba, Luz StellaSantos-Gomes, GabrielaGómez, F.Santucciu, CinziaGómez, FernandoSapountzis, PanagiotisGómez, Mónica GandíaSaraiva, MarciaGómez-Barrero, SusanaSaratale, Ganesh DattatrayaGomez-Escribano, Juan-PabloSardaro, Maria Luisa SavoGómez-Mejia, AlejandroSaribaş, Abdullah SamiGómez-Muñoz, María TeresaSarkar, AbhijitGomez-Silva, BenitoSarkar, AmritaGomis-Cebolla, JoaquinSarkar, AnujitGomoryova, ErikaSarmiento, CeciliaGonçalves, Ana L.Sarmiento, LuisGonçalves, PaulaSaro, CristinaGonçalves-Pereira, JoãoSarshar, MeysamGoncharenko, Anna V.Sarwar, ZaaraGonda, SándorSateriale, AdamGondek, MichałSathiyaraj, SrinivasanGonella, ElenaSatish, LakkakulaGong, BinSato, HiroshiGong, Gwo-chingSato, Marcello OtakeGonzalez, Juan M.Satta, GiovanniGonzález-Candelas, LuisSaviano, AngelaGonzález-Cerón, LiliaSavvidou, Maria G.González-Fandos, María ElenaSawada, KozueGoodfellow, LauraSawers, R. GaryGoodrich-Blair, HeidiSayans, Mario PerezGoormaghtigh, FrédéricSayed, Ibrahim M.Gopal, PramodSazonova, Margarita A.Gorasia, DhanaScaglione, Frine EleonoraGordeliy, ValentinScalschi, Loredana MariaGormley, EamonnScamporrino, Andrea AntoninoGórniak, DorotaScarpellini, EmidioGorny, AdrienneScaturro, MariaGorrasi, SusannaSchaer, JulianeGorres, Kelly L.Scheres, JacquesGórska, Ewa BeataSchill, Kristin MGorski, LisaSchindler, DanielGosciniak, GrazynaSchmacht, MaximilianGosling, Rebecca J.Schmidl, DoreenGospodarek, JaninaSchmidt, HerbertGosselin, MichelSchmidt, MartinGoswami, AnweshaSchmitt, EmmanuelleGötz, Klaus-PeterSchmitz, Jonathan E.Goudarzi, MaryamSchnierle, Barbara S.Gougoulis, Dimitris A.Schoenrock, KathrynGould, Odette N.Schriner, Samuel E.Gould, PhillipSchröttner, PercyGowans, EricSchubert, KristinGoyer, ClaudiaSchuren, FrankGozdowski, DariuszSchütte, KerstinGrabarczyk, MałgorzataSchuurman, Henk-JanGrabau, Larry J.Schwalm, Nathan D.Gradissimo, AnaSchwan, WilliamGragg, Sara E.Schwartz, ChristianGralak, Mikołaj AntoniSchwarz, PatrickGramolelli, SilviaSchwarzer, RolandGranato, MarisaScorpio, DianaGrande, RossellaScott, DanielGrangeteau, CedricScrascia, MariaGranica, SebastianScribano, DanielaGrass, GregorSeal, BruceGrassi, AusiliaSebbane, FlorentGreen, David H.Secchi, EleonoraGreen, SabrinaSedlar, KarelGregori, AlbertoSegovia, CristopherGreif, GonzaloSeibold, Gerd MichaelGrenga, LuciaSekhavati, Mohammad HadiGreses Huerta, SilviaSeleiman, Mahmoud F.Grier, Jennifer T.Selim, Abdelfattah M.Griga, MiroslavSellner, JohannGrippi, FrancescaSelzer, PaulGrishkan, IsabellaSeminari, ElenaGrose, JulianneSen, KeyaGross, RichardSen, Monokesh KumerGrudlewska-Buda, KatarzynaŞenilă, MarinGruffat, HenriSenpuku, HidenobuGruzdkov, Andrei AndreevichSeravalle, GinoGrygier, AnnaSereno, DenisGrygorcewicz, BartłomiejSergeeva, Oksana A.Grywalska, EwelinaSergkelidis, DaniilGrzesiak, JakubSerov, PavelGsaller, FabioSerpico, RosarioGu, LiuqiSerra, RaffaeleGualerzi, Claudio O.Serrano, IreneGuan, JiewenSerrapica, FrancescoGuard, JeanSerwer, PhilipGuembe, MaríaŠešelja Perišin, AnaGuérin, FrançoisSessa, LuciaGuerre, PhilippeSessa, RosaGuerrero, Sergio A.Šestanović, StefanijaGuirao-Abad, José PedroSettanni, LucaGuisado-Vasco, PabloSeubert, Erica L.Güldiken, NurdanSeung-Hun, LeeGullon, BeatrizSevilla-Morán, BeatrizGülses, AydinSha, ShaGunaje, JayaramaShanahan, FergusGuo, BingSharafutdinov, IrshadGupta, KuldeepSharma, DishaGupta, YashSharma, MansiGursky, VitalySharp, Claire R.Gurzawska, KatarzynaSharry, John MacGutierrez, ClaudeShaw, AndrewGutiérrez, Juan CarlosShchyogolev, SergeiGutiérrez-Gutiérrez, CarlosShearwin, Keith E.Gutiérrez-Martin, César B.Shelake, Rahul MahadevGuy, Rebecca A.Shelomi, MatanGuzmán-Novoa, ErnestoShemesh, MosheGuzzon, RaffaeleShen, Ching JuGweon, Hyun SoonShen, JianxunGwiazdowska, DanielaShen, Ming-YiGyöngyvér, MaraSheorey, HarshaHa, Nam-ChulShepelkova, GalinaHaataja, SauliSherer, NathanHać-Szymańczuk, ElżbietaShestopalov, Alexander MHaddad, NabilaShi, HaoshenHadziyannis, EmiliaShiao, Shin-HongHaese, Nicole N.Shih, Chun-KuangHaeseler, Arndt VonShikanai-Yasuda, Maria AparecidaHagen, FrickmannShimizu, NaotoHagen, Ralf MatthiasShimohata, TakaakiHahn, AlexanderShimosato, TakeshiHahn, MatthiasShin, Hye JinHajdú, EditShin, Ok SarahHalenius, AnneShinozaki, YukikoHalim, MurniShiryaev, Anton G.Halvatsiotis, Panagiotis G.Shmakov, Sergey A.Hammerl, Jens AndréShort, BrynHan, Dong-WookShringi, SmritiHang, JunShtark, Oksana Y.Hanga, Mariana PetronelaShukla, DeepakHankaniemi, Minna M.Shukla, Pushp SheelHann, Hie-WonShumkova, RositsaHanson, LisaSiah, AliHao, JihuaSiddiqui, ArifHao, JinsongSidiropoulou, ErasmiaHao, Zhi-MinSidorenko, SergeyHardies, Stephen C.Šilha, DavidHardtke-Wolenski, MatthiasSilva, EdsonHarl, JosefSilva, GoncaloHarman, GarySilva, Marcelo SousaHarnisz, MonikaSilvestre, AnneHarrach, BalázsSilvestrini, LuciaHarris, David T.Simitzis, PanagiotisHartmann, AntonSimões, João Carlos CaetanoHaruki, MitsuruSimões, ManuelHasan, Nabeeh A.Simon, ArneHasan, ShakirSimonetti, GiovannaHasegawa, MakotoSimonini, RobertoHashimoto, MasayukiSimpson, David J.Hasni, IssamSingh, GatikrushnaHassan, Saad El-DinSingh, MahimaHassan, Sherif T.S.Singh, RajbirHassani, Mohamed-AmineSingh, RaveshHatamoto, MasushiSingh, ReemaHatfull, GrahamSingh, SnehaHauffe, HeidiSingh, SukhvinderHawman, DavidSinha, DhritiHay, RoderickSiniagina, MariaHayashi, HidenoriSinigaglia, AlessandroHayashida, KenjiSiniscalco, DarioHayes, Sidney J.Sionov, RonitHe, LixiaSirobhushanam, SirishaHeczko, PiotrSitkowska, BeataHeersema, Lara A.Siupka, PiotrHeinisch, Jürgen J.Sivalingam, PeriyasamyHeinonen, Krista M.Sjöqvist, ConnyHelle, FrancoisSkaradzińska, AnetaHelmby, HelenaSkerlavaj, BarbaraHelton, Rebekah R.Skliros, DimitriosHenke, Nadja AlinaSkoglund, ErikHensel, MichaelSkorko-Glonek, JoannaHerbein, GeorgesSkovgaard, OleHerbort, Carl PSkowronek, MarcinHeredero-Bermejo, IreneSkrypnik, KatarzynaHernández Núñez, EmanuelSkrypnik, Liubov N.Herosimczyk, AgnieszkaSkrzypczak, KatarzynaHerrera, DavidSkvarč, MihaHerreweghen, Florence VanSlaná, IvaHesson, JennySlate, AnthonyHetényi, CsabaŠmajs, DavidHeyndrickx, MarcSmedile, FrancescoHidaka, MichioSmith, KerryHijano, Diego R.Smolander, Olli-PekkaHijri, MohamedSmolen, Kinga K.Hilbert, FriederikeSmucker, NathanHildebrandt, Jan-PeterSmulski, SebastianHilgarth, MaikSneh, BaruchHinkula, JormaSnini, SelmaHirai, ItaruSnyder, LoriHirai, NobumitsuSoares, FabianaHirose, YuuSoares, HelenaHo, Cheng-MawSoares-Castro, PedroHo, ChichunSocea, BogdanHo, LongSoeters, Heidi M.Ho, Tzu-ChuanSoffritti, IreneHoagland, DaisySofia, MarinaHocart, CharlesSogorb Sánchez, Miguel ÁngelHofer, AndersSolano, FranciscoHolatko, JiriSolem, ChristianHolb, ImreSolís García Del Pozo, JuliánHolbrook, MichaelSoltys, KatarinaHole, Camaron RSombetzki, MartinaHolík, LadislavSomeya, NobutakaHolmager, PernilleSommerhalter, MonikaHomblé, FabriceSong, JeongminHong, BangxingSong, Moon JungHong, Bo-YoungSoo Ko, KwanHong, Chang PyoSoreq, LilachHong, Jiann-RueySorrentino, E.Hong, JinsuSossidou, Evangelia N.Hönig, VáclavSoti, Pushpa G.Hopken, Matthew W.Soto, EstebanHõrak, RitaSoto, ManuelHorel, ÁgotaSourimant, JulienHorhat, Florin GeorgeSozhamannan, ShanmugaHori, KatsutoshiSpeciale, ImmacolataHornung, BastianSpeijer, DaveHoshina, TakayukiSpengler, Jessica R.Hosomi, KojiSpennati, FrancescoHotos, George N.Spidlova, PetraHou, Chih-YaoSpiers, AndrewHoughton, Karen MSpiliopoulou, AnastasiaHouhoula, DimitraSpina, FedericaHoward, JonathanSpiridonov, SergeiHoyne, Gerard FSpychala, MarcinHrabar, JerkoSrivastava, AnkitaHrdy, JiriSrivastava, MugdhaHsiao, AnselSrivastava, Rajiv RanjanHsieh, Feng-ChiaStachyra, AnnaHsu, Bing-MuStadler, MarcHsu, Jih-TayStadnyk, AndrewHsu, Pang-HungStaehelin, ChristianHsu, Wen-LiStammler, GerdHu, ChanglingStan, LauraHu, JieStandley, Claire JHu, MengjunStanford, KimHu, QingStarič, JožeHuang, BoStarrett, Gabriel J.Huang, ChiachiStasiak-Różańska, LidiaHuang, Chien-JuiStäubert, ClaudiaHuang, Fu-ChenStchigel, Alberto MiguelHuang, Guan-JhongStear, MichaelHuang, HaoStefanini, IreneHuang, I-HsiuStein, LisaHuang, KangSteinberg, DoronHuang, LiliStein-Thoeringer, ChristophHuck, OlivierStendahl, OlleHudson, Bernard J.Stepanova, EkaterinaHulme, JohnStephan, RogerHurst, Brett L.Stępień, ŁukaszHurst, Jerod J.Stępień-Pyśniak, DagmaraHussain, MubsharSter, CélineHutinet, GeoffreySterer, NirHuyben, DavidSterken, MarkHynes, Michael F.Steven, Blaire T.Hyrsl, PavelStevens, Ann M.Iannelli, FrancescoStewart, Christopher J.Ianni, AndreaStiborova, HanaIbaba, Jacques DavyStilianakis, Nikolaos I.Ibrahim, AhmedStilwell, PeterIbrahim, BarrakStingl, KerstinIchihashi, NorikazuStingu, Catalina-SuzanaIglesias, M. José GarcíaStoyanova, AlbenaIgnatov, AlexanderStoyneva-Gärtner, MayaIkeh, MélanieSt-Pierre, BenoitIlinykh, Philipp A.Straub, ChristinaIlyas, SadiaStrbak, OliverIlyasov, RustemStres, BlažIm, JeongdaeStriedner, GeraldIm, Syn-HeyogStrle, FrancInês, AntónioStrzałka, KazimierzIngel, BrianStrzelecki, JanuszIngham, Colin J.Stupica, DašaIngle, HarshadSuárez, Cinta PrietoInomura, KeisukeSuárez-Estrella, F.Inoue, JunSubbiah, JeevaIntra, JariSubramanian, SowmyalakshmiInturri, RosannaSuchodolski, JakubIordanskiy, SergeySue, Shih-CheIorizzo, MassimoSuhrbier, AndreasIost, IsabelleSujkowska-Rybkowska, MarzenaIovino, FedericoSulej, JustynaIqbal, Hafiz M. N.Sulo, PavolIrinyi, LaszloSummers, Katie LynnIrla, MartaSundberg, Eric J.Iseppi, RamonaSunna, AnwarIslam, JahidulSunpapao, AnuragIslam, Md TohidulSuperti, FabianaIslam, RabiulSurana, PriyankaIsmaiel, AdnanSuriano, FrancescoIsoda, NorikazuSurup, FrankIsokpehi, RaphaelSutton, GrangerIsola, DanielaŠvedienė, JurgitaIturri, JagobaSvetec Miklenić, MarinaIvanov, MarijaSvircev, AntonetIvanova, Anastasia A.Swei, AndreaIvanova, Olga E.Świątek, ŁukaszIvask, AngelaŚwiątkiewicz, MałgorzataIvshina, IrenaSwiecilo, AgataIwagami, MoritoshiSydow, MateuszIyer-Pascuzzi, Anjali SSynowiec, AgnieszkaIzdebska, Joanna N.Syranidou, EvdokiaIzquierdo-Bueno, InmaculadaSzabo, AttilaIzzetti, RossanaSzacon, ElzbietaJackson, Sarah E.Szafranek-Nakonieczna, AnnaJacobs, MeganSzalewska-Palasz, AgnieszkaJacobs-Sera, DeborahSzandruk, MartaJacqz-Aigrain, EvelyneSzczepankowska, Agnieszka K.Jagielska, ElżbietaSzetpizeowski, JacekJakab, EndreSzkaradkiewicz, AndrzejJakobsen, MogensSzpyrka, EwaJakobs-Schönwandt, DesireeSzteyn, JoannaJakšić, DanielaSzweda, PiotrJakubska-Busse, AnnaSzymanski, ChristineJames, ArnoneTabacchioni, SilviaJames, Claire DTagliavia, MarcelloJames, Euan KevinTahrioui, AliJan, Fuh-JyhTakahashi, IkukoJana, BimalTakashige, KashimotoJang, JeonghwanTakeda, MakotoJang, SoojinTakehiro, NishidaJanowicz, AnnaTakeuchi, RikiyaJansen, Jes La CourTalà, AdelfiaJanusz, GrzegorzTambong, James T.Jarosz-Wilkołazka, AnnaTan, Bee KangJass, JanaTana, ClaudioJaswal, RajneeshTanaka, MasaruJauregui, Rodrigo GutierrezTanase, CorneliuJavaherdashti, RezaTandon, RiteshJaworek, Andrzej KazimierzTang, QiyiJayaraman, SundararajanTanner, N. KyleJeannot, KatyTarasco, EustachioJensen, Per MoestrupTarr, Gillian A. M.Jenssen, HåvardTarrah, ArminJeon, SoheeTassone, SoniaJeong, Rae-DongTatu, Alin LaurenţiuJergovic, MladenTausch, Simon H.Jerônimo, Gabriela TomasTavío, María M.Jesenak, MilosTaylor, MartinJesudoss Chelladurai, JebaTaylor, Richard S.Jeudy, SandraTeaumroong, NeungJeyanathan, JeyamalarTedim, Ana P.Jeżewska-Frąckowiak, JoannaTegetmeyer, Halina E.Ji, ShaofeiTeixeira, PilarJiang, Jhih-HangTeixeira-Santos, RitaJiménez Ballesta, RaimundoTelatin, AndreaJiménez Gómez, Pedro A.Teleky, BernadetteJiménez, CarlosTellez-Lance, AngelaJindo, KeijiTender, Caroline DeJindou, SadanariTenea, GabrielaJohansen, Matt D.Teng, Ching-HaoJohansson, BjörnTeper, DoronJohnson, ElizabethTerzić-Vidojević, AmarelaJohnston-Monje, DavidTesei, DonatellaJonas, Kai J.Tetaud, EmmanuelJones, Cerith A.Tettamanti, GianlucaJones, ChrisTetz, GeorgeJones, George H.Thanki, Anisha MahendraJones, MarjorieTheriot, Casey M.Jones, Rheinallt M.Thiruvengadam, MuthuJoniec, JolantaThomas, Donald B.Jordao, LuisaThomson, RachelJorge, Gomez-GutierrezThorpe, ClareJucan, DenisaThurber, RebeccaJurado, Macarena M.Tian, SonghaiJurasek, MichalTichaczek-Goska, DorotaJurenas, DukasTidona, FlavioJuszczak, LesławTikhe, Chinmay V.Kabasawa, YujiTilocca, BrunoKabeya, NaokiTinker, JulietteKačániová, MiroslavaTiscar, Pietro G.Kaczmarczyk-Ziemba, AgnieszkaTischler, DirkKaczmarek, AgataTisi, RenataKaden, ReneTkaczuk, CezaryKagambèga, AssètaTkadlec, JanKahl, Barbara C.Tkavc, RokKajak-Siemaszko, KatarzynaToczylowska-Maminska, RenataKajsík, MichalTodorov, SvetoslavKaldhone, PravinTodorova, DessislavaKalenik, MarekTofalo, RosannaKalicińska, ElżbietaTogawa, AtsushiKállai, ZoltánTołkacz, KatarzynaKalra, DivyaTomaszewska, EwaKalsi, MeghaTonelli, MicheleKałucka, IzabelaToniolo, AntonioKalyuzhnaya, MarinaToporowska, MagdalenaKamath, KarthikTorres, Marcelo Der TorossianKambourova, MargaritaTorres-Collado, LauraKaminski, Tomasz W.Torresi, MarinaKampf, GünterTorres-Maravilla, EdgarKandel, Prem PrasadTorri, EmanueleKandylis, PanagiotisTorta, LivioKanematsu, HideyukiTorzewska, AgnieszkaKanetis, LoukasTosi, MicaelaKanipe, CarlyTouceda-González, MaríaKaňková, ŠárkaTouret, FranckKao, Cheng-YenTourlousse, DieterKapellos, George E.Town, JenniferKaplan, AaronTownsend, EleanorKaplan, ShaunaTraina, GiovannaKapusta, PiotrTrček, JanjaKaraduta, OlegTripathi, AnupriyaKarama, MusafiriTrippe, KristinKaranis, PanagiotisTrögl, JosefKaraś, MagdalenaTrombetta, ElenaKaraüzüm, HaticeTroyer, RyanKarbownik, Michal S.Trucillo, PaoloKardos, GáborTruncaitė, LidijaKarisola, PiiaTrzciński, KrzysztofKarkowska-Kuleta, JustynaTsai, Kun-HsienKarpiński, Tomasz M.Tsaltas, DimitrisKarunarathna, Samantha C.Tseng, Jen-ChihKarus, AvoTsouknidas, AlexanderKarwaciak, IwonaTsuchida, SachioKarwowski, BoleslawTuchscherr, LorenaKashiwagi, AkikoTumban, EbenezerKasperkiewicz, KatarzynaTun, Mya Myat NgweKaszowska, MartaTung, Yen ChenKatada, KazuhiroTunsjø, HegeKatapodis, PetrosTuomanen, ElaineKathare, Praveen KumarTurner, ClaireKathayat, DipakTveit, Alexander TøsdalKatinakis, PanagiotisTvrdá, EvaKato, HideoTylicki, LeszekKato, HirotomoTyurin, Anton P.Kato, JunichiTzeng, Shiang-JongKato, YusukeTzortzakis, NikosKatsifas, Efstathios A.Ucciferri, ClaudioKauffman, KathrynUda, AtsushiKavita, KumariUngureanu, NicoletaKavousi, JavidUpadhyay, SandeepKawabata, KyuichiUrbanowska, AgnieszkaKawalek, AdamUrbonavičius, JauniusKawasaki, KiyonoriUrrutia, HomeroKayali, GhaziVacca, MircoKayumov, AiratVaccarezza, MauroKazimírová, MariaVach, KirstinKazunori, MatsudaVadkertiová, RenátaKędzierska, BarbaraValat, Charlotte.Keiper, Brett D.Valik, LubomirKelarakis, AntoniosValitutti, FrancescoKeller, LanceValm, Alex M.Keller, PeterVamanu, EmanuelKellogg, ChristinaVan Den Berg, Marco AlexanderKelly, GavinVan Dyke, MichaelKemenesi, GáborVan Grevenynghe, JulienKenna, Dervla T.D.Van Hullebusch, Eric D.Kenzaka, TsuneakiVan Kaer, LucKerou, MelinaVan Straalen, Nico M.Kerrouche, AbdelfatehVanderpas, JeanKhalid, HattafVanlandingham, DanaKhan, Aman UllahVargas Guzman, Horacio AndresKhan, FazlurrahmanVargas-García, María Del CarmenKhan, MerajuddinVari, Camil-EugenKhatun, AminaVarrella, StefanoKheimar, Ahmed M.Varsaki, AthanasiaKhmelenina, Valentina N.Vashist, SandeepKhor, Wei ChingVasilchenko, AlexeyKhoury, ZaidVasile, FrancescaKiefer, DorotheeVasina, Daria V.Kieliszek, MarekVassilev, NikolayKiiker, RiinuVazquez Belda, BeatrizKilk, KalleVázquez-Ucha, Juan C.Kim, Bong-SooVeerapandian, RajaKim, Byoung SikVelada, IsabelKim, Doo-JinVelay, AurélieKim, HeejungVelazquez-Salinas, LauroKim, Hye OneVélez, MariselaKim, Jae-SeokVelicer, Gregory J.Kim, Ji HyungVelin, DominiqueKim, Jin-CheolVella, Filomena MonicaKim, Kil-SooVengušt, ModestKim, Ki-YoungVenken, Koen J.T.Kim, Kyoung-HoVerdenelli, Maria CristinaKim, Kyung-MinVergara, AnderKim, Min JungVergine, MarziaKim, Sae-HunVernoux, Jean-PaulKim, SunahVeronico, PasquaKim, Young-ChanVetukuri, RameshKimeklis, Anastasiia K.Viana, FlaviaKindrachuk, JasonViana, Sofia Andreia DominguesKinthada, LakshmanakumarVictor, EmmanuelKirchberger, PaulVidican, RoxanaKirienko, NatashaVidyarathna, Nayani K.Kirkham, Lea-AnnViegas, CarlosKissoudis, ChristosVieira Baptista, PedroKita, DaichiVijayan, MadhuvanthiKitao, TomoeVikram, AmitKittl, SonjaVilela, AliceKlassen, RolandVilla Serrano, MariaKlaumann, FranciniVilla, LucaKlein-González, NelaVillaverde, JaimeKlewicka, ElzbietaVillinger, JandouweKlibi, NaouelVimercatti, LaraKlimek-Ochab, MagdalenaVinardell, José M.Klitz, WilliamVishnyakov, Innokentii E.Klocperk, AdamVisioli, GiovannaKlymiuk, IngeborgVismarra, AliceKmiec, DorotaVitale, FabrizioKmieć, KatarzynaVitale, LucaKobae, YoshihiroVitti, AntonellaKobayashi, KazuoVlainić, JosipaKobayashi, NobumichiVolz, AsisaKobierzycki, ChristopherVon Fricken, MichaelKobiler, OrenVonaesch, PascaleKoblizek, MichalVorobyev, ArtemKoch, LotharVoronina, O. L.Kochanowski, KarlVoulgari-Kokota, AnnaKocot, AleksandraVrdoljak Tomaš, AnaKocsis, BelaVriet, CécileKoebnik, RalfVrioni, GeorgiaKoh, HidefumiVučurović, AnaKoide, Roger T.Vulturar, RomanaKoim-Puchowska, BeataVyatkina, Kira V.Kojic, MilanWagley, SariqaKokkinakis, Emmanouil N.Wałecka-Zacharska, EwaKokkinos, Nikolaos C.Walker, Alejandro R.Kokoszyński, DariuszWalker, JenniferKolář, MilanWallace, RhiannonKolditz, MartinWang, Fun-InKolekar, PandurangWang, HaitaoKoliarakis, IoannisWang, MinKollipara, AvinashWang, PenghaoKolmeder, CarolinWang, ShaoboKomaki, HisayukiWang, Shao-HungKominek, JacekWannicke, NicolaKomínek, PetrWdowiak-Wróbel, SylwiaKomova, AnastasiaWee, JosephineKończak, MagdalenaWegrzyn, GrzegorzKonopacki, MaciejWeidmann, ManfredKonopelski, PiotrWeiland-Bräuer, NancyKonteles, SpyrosWeinrick, Brian C.Kontsevaya, IrinaWemheuer, BerndKooistra, Wiebe H.C.F.Werten, SebastiaanKopernik, MagdalenaWertz, Philip W.Kopp, JulianWhisnant, Adam W.Köppen, KristinWhite, JamesKopprio, GermánWidemann, EmilieKoraimann, GüntherWiegand, SandraKorf, ImkeWielbo, JerzyKorzekwa, KamilaWietrzyk-Pełka, PaulinaKorzeniewska, EwaWiktorczyk-Kapischke, NataliaKorzhenkov, A. A.Wildeboer-Veloo, A.C.M.Kos, BlaženkaWilgan, RobinKosakyan, AnushWilk, MateuszKoschorreck, KatjaWilkinson, DerekKosik-Bogacka, DanutaWillett, JuliaKosmidis, ChrisWilletts, AndrewKostov, GeorgiWilliams, BarbaraKot, Anna MariaWilliams, Rohan B. H.Kot, KarolinaWilliamson, ChristopherKotanidou, AnastasiaWilson, James WKotha, Raghavendhar R.Windshügel, BjörnKotlowski, RomanWińska, KatarzynaKotta-Loizou, IolyWitkowska-Zimny, MalgorzataKotula-Balak, MałgorzataWitt, MartinKoukkou, Anna-IriniWitte, AngelaKouokam, J. CalvinWłodarczyk, MarcinKourkoutas, IoannisWojnicz, DorotaKouvelis, Vassili N.Wojtyczka, RobertKovács, Árpád FerencWolf, Patricia G.Kovacs, RenatoWolfe, Alan JKovács, TamásWolfinger, Michael ThomasKovbasnjuk, OlgaWolny-Koładka, KatarzynaKowalczyk, PawełWong, Chen SeongKowalski, RadosławWoods, Jon P.Kozak, AnnaWoolsey, Ian DavidKožárová, IvonaWorwag, MałgorzataKozdrój, JacekWöstemeyer, JohannesKozieł, EdmundWozniak-Knopp, GordanaKoziróg, AnnaWoźniakowski, GrzegorzKozłowska, MariolaWróbel-Kwiatkowska, MagdalenaKraak, BartWu, ChaoKrabel, DorisWu, Ching-YiKranz, AngelaWu, ChungChunKrawczyk, BeataWu, LinaKremer, DavidWu, Whei-FenKreth, JensWyckoff, KristenKrezel, AndrzejWyczarska-Kokot, JoannaKrier, FrançoisWysok, BeataKrisch, JuditWyszkowska, JadwigaKrischek, CarstenXimenes, EduardoKrishnakumar, DevadasXu, JianpingKrishnasamy, GopinathXu, JingjingKritikos, AntoniosXu, JintaoKrivoruchko, AnastasiiaXu, JunKrockenberger, MarkYamamoto, KyosukeKroeker, AndreaYamamoto, ShingoKról, JolantaYamamoto, TakenobuKrstin, LjiljanaYang, Chul-SuKrug, LaurieYang, Hung-ChiKrumbholz, AndiYang, KunKrumova, SashkaYang, QingKrzebietke, Sławomir JózefYarullina, DinaKrzysztof, AnuszYen, Feng-LinKrzyżek, PawełYeom, JinkiKubiak, KatarzynaYessoufou, AkadiriKucharski, JanYoda, KazutoyoKuczyńska-Wiśnik, DorotaYokomi, RayKuever, JanYoon, WonsuckKufner, VerenaYoshida, NaotoKuhn, RamonaYu, LigenKui, HuangYu, Ollie YiruKulakovskaya, Tatiana V.Yukl, Erik T.Kulkarni, DhananjayaYun, Cheol-WonKulshreshtha, GarimaYusuf, ErlanggaKumar, AjayZaborowska, MagdalenaKumar, AmitZachwieja, AndrzejKumar, AnoopZago, MiriamKumar, GokhleshZaha, Dana CarmenKumar, HirdeshZaheer, AhmadKumar, PrasunZaheer, RahatKumar, RupeshZahmanova, Gergana G.Küppers, Gabriela CristinaZaleśny, GrzegorzKusada, HiroyukiZalewska, MagdalenaKutkowska, JolantaZamorska, JustynaKutsar, KuuloZamperin, GianpieroKuyl, Antoinette C. Van DerZanchi, CarolineKuźniar, AgnieszkaZanfirescu, AncaKvitko, BrianZasloff, MichaelKwak, DongmiZavašnik, JanezKwambana-Adams, BrendaZawrotniak, MarcinKwapisz, MartaZdarta, AgataKwarciak-Kozłowska, AnnaZdrazilova‐Dubska, LenkaKwiatkowski, PawelZębek, ElżbietaKwon, Hyuk-JoonZeineldin, MohamedKwon, Oh-DeogZervas, AthanasiosKyaw Thu, AungZhang, Baiyu (Helen)Kyker, SarahZhang, ChunyeKyriazakis, IliasZhang, ErshuaiKyrkou, IfigeneiaZhang, FanL. Mickol, RebeccaZhang, HuanminLa Porta, RaffaeleZhang, JianhuaLa Scola, BernardZhang, JiaweiLa Spina, MichelangeloZhang, JileiLaabei, MaisemZhang, LeLabudda, MateuszZhang, PeipeiLaca Pérez, AmandaZhang, RuiyongLachenmeier, Dirk W.Zhao, JinxinLahner, EdithZhao, LinboLai, OlimpiaZhu, ShiweiLakatos, TamásZiarno, MałgorzataLalak-Kańczugowska, JustynaZibaee, ArashLalonde, LauraZielińska, DorotaLamas, AlexandreZieliński, MarcinLami, RaphaëlZieniuk, BartłomiejLamien Meda, AlineZimowska, BeataLandell, MelissaZingl, FranzLandi, LuciaZito, GabriellaLang, AndrewZlatian, OvidiuLanga, SusanaZmora, PawelLanger, Gitta JuttaZühlke, DanielaLangridge, GemmaZulkharnain, AzhamLanneluc, IsabelleZúñiga, ManuelLantieri, FrancescaZur, JoannaLapaquette, PierreZüst, RolandLaplace, Jean-Marie



